# Case report: Twice-daily tolvaptan dosing regimen in a challenging case of hyponatremia due to SIAD

**DOI:** 10.3389/fendo.2023.1309657

**Published:** 2024-01-15

**Authors:** Anna Arecco, Davide Demontis, Leonardo Della Sala, Natale Musso, Stefano Gay, Mara Boschetti, Diego Ferone, Federico Gatto

**Affiliations:** ^1^ Endocrinology Unit, Department of Internal Medicine and Medical Specialties, School of Medical and Pharmaceutical Sciences, University of Genova, Genova, Italy; ^2^ Endocrinology Unit, IRCCS Ospedale Policlinico San Martino, Genova, Italy

**Keywords:** syndrome of inappropriate antidiuresis, hyponatremia, vasopressin receptor antagonists, tolvaptan, sodium, corpus callosum agenesis, frontal teratoma

## Abstract

**Background:**

Syndrome of inappropriate antidiuresis (SIAD) is one of the most frequent causes of euvolemic hyponatremia (serum sodium levels < 135 mEq/L) and it represents more than 35% of hyponatremia cases in hospitalized patients. It is characterized by an inappropriate vasopressin (AVP)/antidiuretic hormone (ADH) secretion, which occurs independently from effective serum osmolality or circulating volume, leading to water retention via its action on type 2 vasopressin receptor in the distal renal tubules. Corpus callosum agenesis (CCA) is one of the most common congenital brain defects, which can be associated to alterations in serum sodium levels. This report presents a rare case of chronic hyponatremia associated with SIAD in a woman with CCA, whose correction of serum sodium levels only occurred following twice-daily tolvaptan administration.

**Case presentation:**

A 30-year-old female was admitted to our hospital for non-acute hyponatremia with dizziness, headache, distal tremors, and concentration deficits. She had profound hyponatremia (Na 121 mmol/L) with measured plasma hypo-osmolality (259 mOsm/Kg) and urinary osmolality greater than 100 mOsm/Kg (517 mOsm/Kg). She presented clinically as normovolemic. After the exclusion of other causes of normovolemic hyponatremia, such as hypothyroidism and adrenal insufficiency, a diagnosis of SIAD was established. We have ruled out paraneoplastic, inflammatory, and infectious causes, as well as ischemic events. Her medical history showed a CCA and frontal teratoma. We administered tolvaptan initially at a low dosage (15 mg once a day) with persistence of hyponatremia. Therefore, the dosage was first doubled (30 mg once a day) and then increased to 45 mg once a day with an initial improvement in serum sodium levels, although not long-lasting. We therefore tried dividing the 45 mg tolvaptan administration into two doses of 30 mg and 15 mg respectively, using an off-label treatment schedule, thus achieving long-lasting serum sodium levels in the low-normal range associated with a general clinical improvement.

**Conclusions:**

This report underlines the importance of the correct diagnosis, management and treatment of SIAD, as well as the need for further studies about the pharmacokinetics and pharmacodynamics of vasopressin receptor antagonists.

## 1 Introduction

The syndrome of inappropriate antidiuresis (SIAD), first described by Bartter and Schwartz in 1957 (1), is one of the most frequent causes of hyponatremia. It represents more than 35% of hyponatremia cases in hospitalized patients (2). SIAD is characterized by an inappropriate vasopressin (AVP)/antidiuretic hormone (ADH) secretion, which occurs independently from effective serum osmolality or circulating volume. An increased synthesis by the hypothalamus and consequent release by the posterior pituitary gland, or the presence of paraneoplastic syndromes, may be possible causes. Moreover, a gain of function mutation in the type 2 vasopressin receptor (V2) may lead to inappropriate antidiuresis (3). Several clinical conditions have been associated to inappropriate antidiuresis: among them, the most common are cancers, pulmonary diseases and central nervous system disorders (3).

The clinical presentation is usually not specific and paucisymptomatic due to its slowly progressive onset. Gait instability, dizziness, falls, concentration and cognitive deficits, headache, and nausea with or without vomiting are the most common symptoms. Lastly, hyponatremia is associated with an increased risk of death (4). SIAD is a diagnosis of exclusion and needs the presence of all essential criteria. Alternatively, additional criteria may help making a correct diagnosis of SIAD (**Table 1**) (6). Fluid restriction is the cornerstone in correcting non-hypovolemic chronic hyponatremia (6). Other approaches include increased osmotic intake, such as urea (7, 8) or sodium chloride (NaCl) tablets (9). The non-peptide vasopressin V2 receptor antagonists represent a valuable strategy for the treatment of SIAD (10, 11). FDA approved first conivaptan and later tolvaptan for treating chronic hyponatremia associated with heart failure, liver cirrhosis, and SIAD (12). EMA approved only tolvaptan for treating hyponatremia due to SIAD (13). Tolvaptan is administered orally once a day, as for EMA information leaflet (14).

**Table 1:** Diagnostic criteria for the syndrome of inappropriate antidiuresis.

Essential criteria
Effective serum osmolality < 275 mOsm/kg
Urine osmolality > 100 mOsm/kg at some level of decreased effective osmolality
Clinical euvolemia
Urine sodium concentration > 30 mmol/L with normal dietary salt and water intake
Absence of adrenal, thyroid, pituitary or renal insufficiency
No recent use of diuretic agents
Supplemental criteria
Serum uric acid < 0.24 mmol/L (< 4 mg/dL)
Serum urea < 3.6 mmol/L (< 21.6 mg/dL)
Failure to correct hyponatremia after 0.9% saline infusion
Fractional sodium excretion > 0.5%
Fractional urea excretion > 55%
Fractional uric acid excretion > 12%
Correction of hyponatremia through fluid restriction

Adapted from Schwartz WB et al. (1) and Janicic N & Verbalis JG (5).

Corpus callosum agenesis (CCA) denotes the absence of the main commissural fiber white matter tracts that connect the cerebral hemispheres. It may be complete or partial when only the posterior segments of the corpus callosum are missing (15). It has a prevalence of 1:4000 to 1:5000 newborns and it represents one of the most common congenital brain defects (16). Symptoms vary from severe to absent, and it may present with cognitive deficits, muscle tone impairments and neuropsychological disorders (17). Alterations in serum sodium levels have previously been described in association with CCA. Hypernatremia due to central diabetes insipidus (18) has been described in the context of septo-optic dysplasia, characterized by hypoplasia of the optic nerve, agenesis of the septum pellucidum and corpus callosum and hypoplasia of the hypothalamic-pituitary axis (19). Hyponatremia associated with SIAD has been described in infants (20), children (21, 22) and adults (23).

Intracranial teratomas represent only about 0.5% of all intracranial tumors. Based on histopathological evaluation, teratomas are divided into mature, immature and malignant types. Mature teratomas are composed of well-differentiated elements, whereas immature teratomas contain components resembling fetal tissues (24). Teratomas usually originate near the midline or from the pineal region, while the frontal lobe is a less common site of origin. Symptoms depend mainly on tumor location, and they are relatively mild if the teratoma is mature and grows slowly. An association with neuropsychiatric symptoms has been proven for those originating from the frontal lobe (25).

Herein, we describe the case of a female patient with idiopathic SIAD associated with CCA whose long-lasting correction of serum sodium levels occurred only after moving to a twice-daily tolvaptan administration, instead of the standard once daily regimen. To date, a single study comparing the once and twice-daily tolvaptan dosing regimens in patients with heart failure has been published, and it showed no significant pharmacokinetic and clinical differences using these treatment schedules (26). Our report underlines the need for further studies about the pharmacokinetics and pharmacodynamics of vasopressin receptor antagonists.

## 2 Case description

**Figure 1:**
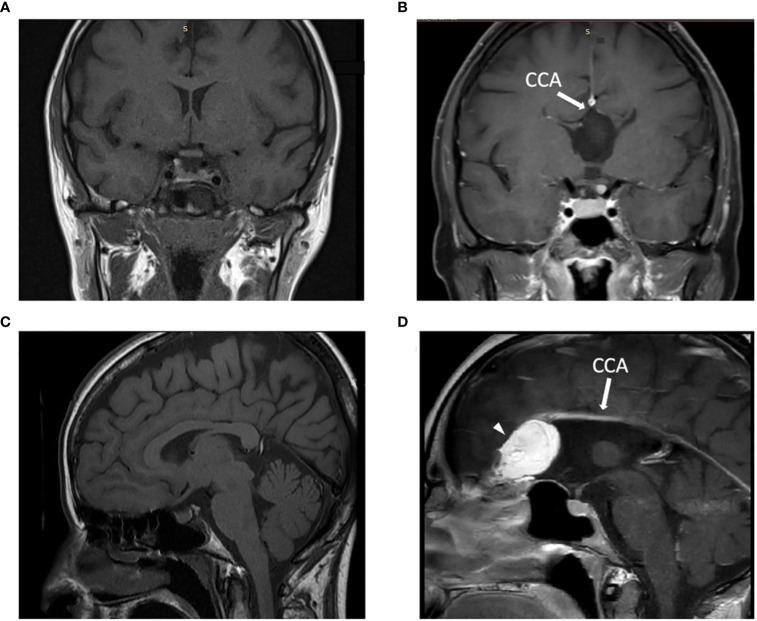
Patient’s MRI imaging and comparison with a control subject: T1-weighted MRI image in the coronal **(A, B)** and in the sagittal **(C, D)** plane demonstrated the presence of the corpus callosum **(A-C)** in a control subject and the corpus callosum agenesis **(B-D)** in our patient, indicated with white arrow. In D an arrowhead indicates a hyperintense lesion on the left frontal lobe consistent with intracranial teratoma. CCA: corpus callosum agenesis. Of note, the control subject is a female patient that performed MRI as follow-up radiological evaluation in the context of a clinical history of non-functioning pituitary adenoma, which underwent successful transsphenoidal neurosurgery.

In September 2022, a 30-year-old female was admitted to our hospital for non-acute hyponatremia. The patient was paucisymptomatic: she reported dizziness, headache, distal tremors, and concentration deficits. Her medical history showed corpus callosum agenesis and frontal teratoma in neuroradiological follow-up (**Figure 1**). No medication was taken apart from antihistamines as needed. No data regarding her previous serum sodium levels were available.

On physical examination, the patient presented as normovolemic: blood pressure was within the normal range (SBP 100 mmHg, DBP 70 mmHg), and no signs of peripheral oedema, pulmonary congestion and mucous membrane dryness were detected. Blood chemistry tests showed profound hyponatremia (Na 121 mmol/L) with measured plasma hypo-osmolality (259 mOsm/Kg) in normal renal function (serum creatinine 0.5 mg/dL with estimated GFR > 90 mL/min), as well as reduced levels of urea (7 mg/dL) and uric acid (2.3 mg/dL). In addition, we appreciated a urinary osmolality greater than 100 mOsm/Kg (517 mOsm/Kg) and spot urinary sodium greater than 30 mEq/L (88 mEq/L). Therefore, we diagnosed chronic hypotonic hyponatremia in the presence of inappropriately concentrated urine associated with normal extracellular fluid volume.

On the assumption of SIAD, further investigations were carried out. We ruled out adrenal deficiency (morning plasma cortisol 9.6 μg/dL, ACTH value of 35.5 ng/L and urinary free cortisol 31.5 μg/24h) and hypothyroidism (TSH 1.57 mIU/L, fT4 17.97 ng/L). The gonadotroph (LH 10.65 U/L, FSH 5.29 U/L, 17-β estradiol 371 ng/L) and somatotroph (IGF-1 144 μg/L, 40^th^ percentile) axes were preserved. We only observed mild hyperprolactinemia (PRL 56.45 μg/L, with a 47% recovery after polyethylene glycol serum precipitation). Inflammatory markers (C-reactive protein < 3 mg/L, erythrocyte sedimentation rate 5 mm/h and procalcitonin 0.02 μg/L) were negative. The diagnosis of SIAD was therefore established.

To rule out paraneoplastic, inflammatory, and infectious causes, we performed a contrast-enhanced computed tomography (CT) imaging of the chest and abdomen, which did not show any clear sign of neoplasia or inflamed areas. Furthermore, there was no evidence of neurological disease, and the brain magnetic resonance imaging (MRI) showed no evidence of neurological injuries or ischemic events. Thus, we determined the idiopathic nature of SIAD.

**Figure 2:**
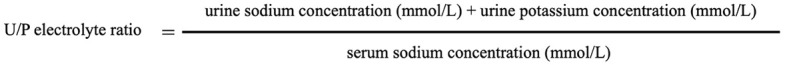
Furst formula. Adapted from Grant et al. (27).

Afterwards, we used the Furst formula (**Figure 2**), which estimates the electrolyte-free water clearance through the urine/plasma electrolyte ratio, obtaining a value > 1.0, which is indicative of reduced electrolyte-free water clearance. Therefore, due to this index and the recorded value of urinary osmolality (> 500 mOsm/kg), we did not start water restriction in our patient, expecting it to be ineffective. However, we initiated treatment with tolvaptan (a non-peptide vasopressin V2 receptor antagonist) at the dosage of 15 mg one tablet once a day.

After an initial improvement of the serum sodium levels, reaching a value of 129 mmol/L just 10 hours after the first drug administration, with a calculated serum osmolality of 265 mOsm/kg, we observed a persistent profound hyponatremia with a nadir of 124 mmol/L. Following the trend of serum sodium levels and the lack of a significant rise of plasma osmolality, we gradually increased the tolvaptan dosage to 45 mg without obtaining a long-term normalization of serum sodium. We attained only two normal serum sodium values using a 45 mg once-a-day dosing regimen, with a zenith of 136 mmol/L. These data were concordant with the calculated plasma osmolality, since we always appreciated a reduced plasma osmolality apart from two values of 278 and 281 mOsm/kg (above the threshold of 275 mOsm/kg, considered one of the essential SIAD diagnostic criteria).

Performing multiple daily evaluations of serum sodium levels, we observed a trend for a partial correction within the first hours after tolvaptan administration, although followed by a decrease during the day. Therefore, we attempted to divide tolvaptan administration into two daily doses (30 mg at 8 am in the morning and 15 mg at 8 pm in the evening, respectively). Interestingly, we witnessed a consequent stabilization of serum sodium levels: at the patient’s discharge serum sodium levels were 133 mmol/L, and at the following controls in the outpatient clinic we observed an electrolyte normalization with serum sodium levels of 138 mmol/L and 136 mmol/L, after two weeks and three months respectively. Moreover, there was a rise in the calculated plasma osmolality, obtaining 283 mOsm/kg and 278 mOsm/kg at the outpatient follow-up visits. Furthermore, we detected a 1.5 Kg body weight loss and a progressive increase in daily urinary volume from 1600 mL to 2000 mL, without changes in water intake, in the days following the tolvaptan dosing regimen modification. At the same time, the patient reported a general improvement in the symptoms complained at the hospital admission.

**Figure 3:**
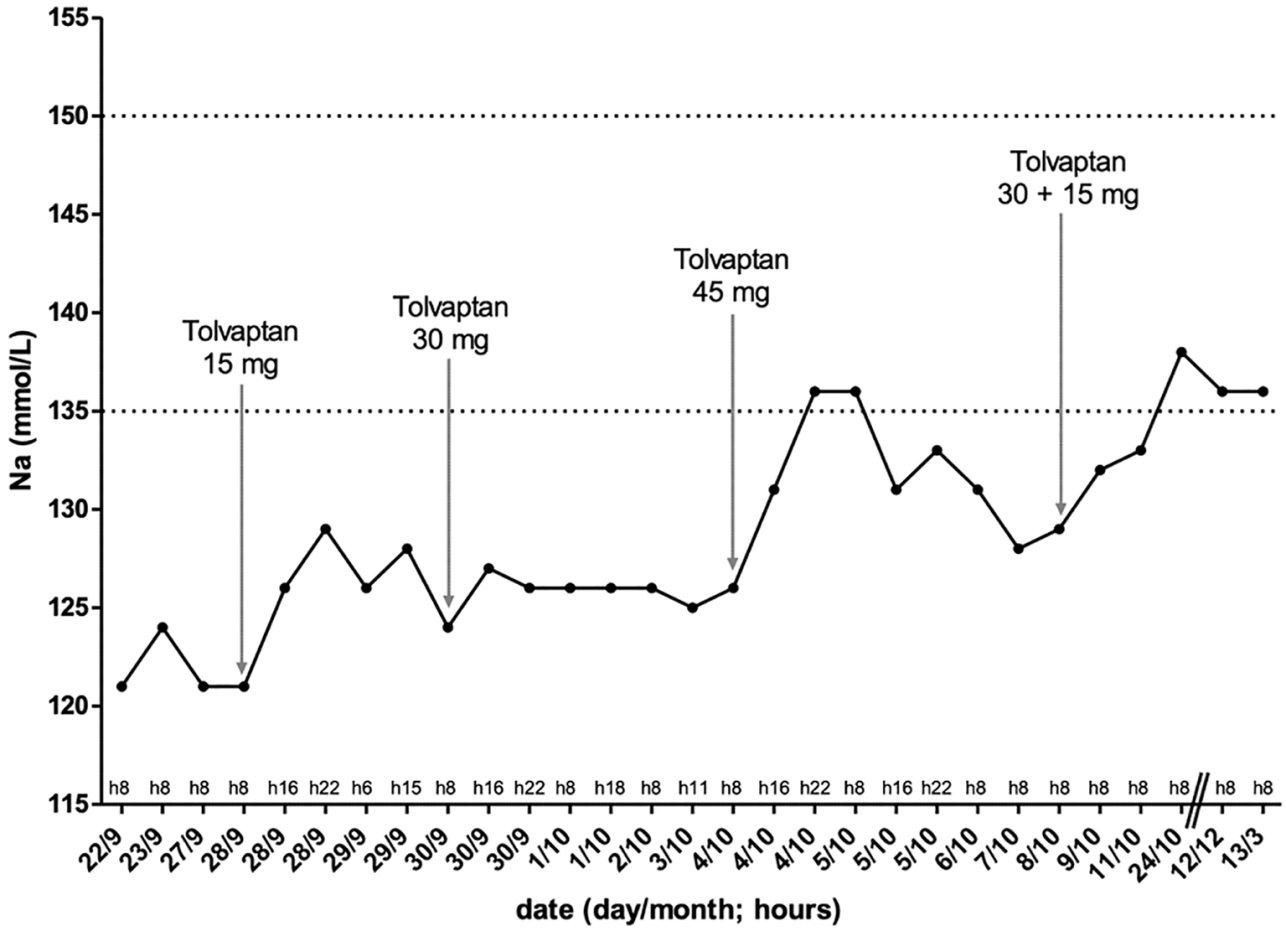
Changes in patient serum sodium levels through the observation period, together with the varying daily dosages of tolvaptan: as tolvaptan dose increased, serum sodium levels increased, but it was only with twice-daily tolvaptan administration that serum sodium levels persisted in the normal range. Na: serum sodium.

**Figure 3** shows the changes in patient’s serum sodium levels throughout the observation period, with the corresponding daily dosages of tolvaptan.

## 3 Discussion

This case report is an interesting example of the challenges met by clinicians in the treatment of hyponatremia due to idiopathic SIAD. According to the latest guidelines (6, 28, 29), the first therapeutic line in chronic hyponatremia is represented by fluid restriction even though the evidence about its higher efficacy in increasing serum sodium levels compared to placebo is sparse. Furthermore, fluid restriction has been recently demonstrated to induce a modest correction of serum sodium levels in a randomized-controlled trial (30). However, clinical experience supports its use if water restriction is strictly adhered to by patients. In order to estimate the degree of restriction necessary it may be helpful the Furst formula, which estimates the electrolyte-free water clearance through the urine/plasma electrolyte ratio (U/P) (**Figure 2**) (27):

- If U/P is < 0.5, start a fluid restriction of 1000 mL/day.
- If U/P is 0.5–1, start a fluid restriction of 500 mL/day.
- If U/P is > 1.0 and in patients with urine osmolalities higher than 500 mOsm/kg H_2_O, there is no excretion of electrolyte-free water and fluid restriction is unlikely to correct hyponatremia.

The patients may not well accept fluid restriction and it is not always useful: actually, Winzeler et al. recently showed that fluid restriction is effective in 59% of patients with SIAD (31). In addition to a Furst index > 1.0, the other possible predictors of nonresponse to fluid restriction are UOsm > 500 mOsm/L and UNa > 130 mmol/L. As reported by Garrahy et al., the withdrawal rate to fluid restriction may be not negligible. Patients have to face the resetting of the thirst threshold, which makes the compliance poorer especially in a medium-long-term setting (30). Based on the patient’s U/P > 1.0, the urinary osmolality > 500 mOsm/kg H_2_O, the patient’s young age and the few symptoms, we decided against water restriction, expecting it to be ineffective.

Medical therapies aimed to increase free water clearance are frequently needed (31). This is the case of urea, which allows an increase in the intake of osmotic solutes and the free water elimination in a practical way. This therapeutic approach is considered a second-line treatment in both the European (6) and American (28) guidelines. Its dosage ranges from 0.25 to 0.50 g/kg per day. A limit is represented by its bitter taste which may reduce the treatment acceptance. To improve patient compliance, it is possible combining urea with flavored liquids to improve its palatability. Other problems are the potential increase of blood urea concentrations and the difficulties in finding this agent (32, 33). In our patient, we had to postpone urea administration because of the difficulties in finding this agent in our country. Alternatively, another way to increase osmol intake is a combination of low-dose loop diuretics and oral sodium chloride.

Another therapeutic approach includes demeclocycline, which derives from tetracyclines and whose action consists in causing a nephrogenic form of diabetes insipidus (34). Appropriate doses of demeclocycline range from 600-1200 mg/die in divided doses. Its side effects include reversible urea increase, infrequent nephrotoxicity and photosensitive skin rash. It is mentioned as a possible therapeutic means in the American (28) and in the British (27) guidelines, whereas the European guidelines don’t recommend its use (6). In our patient, we did not use demeclocycline because it has been withdrawn from the Italian market.

Vasopressin receptor antagonists, also called vaptans, are very interesting treatment options since they inhibit vasopressin receptor activation. They reduce AVP efficacy and inhibit the synthesis and insertion of aquaporin-2 (AQP2) water channels in cells of the collecting duct (35). As a consequence, the number of AQP2 channels is reduced and the renal excretion of solute-free secretion (aquaresis) increases without significantly changing electrolytes excretion (26, 36). FDA approved both conivaptan, which blocks the vasopressin receptor V1a and V2, for euvolemic and hypervolemic hyponatremia in hospitalized patients, and tolvaptan, a selective V2 receptor antagonist, for hyponatremia due to SIAD, heart failure and liver cirrhosis. On the other hand, in Europe only tolvaptan is in trade and EMA approved its use only for SIAD.

Compared to the original AVP, tolvaptan has a 1.8 greater affinity for the V2 receptor and a 29-fold higher selectivity for the V2 receptor compared to the V1 receptor. It has no inhibitory activity at V1b receptors. The starting dose of tolvaptan is usually 15 mg on the first day, and the dose can be raised to 30 mg and 60 mg every 24 hours if serum sodium levels remain <135 mmol/L or the increase in serum sodium levels has been <5 mmol/L in the previous 24 hours. As shown in a real-life study (37), tolvaptan can be administered at lower doses (7.5 mg/die) remaining effective in raising sodium levels, and a dosage 3.75 mg/die is currently under investigation. Another pharmacokinetic study confirmed the validity of the 7.5 mg/die dosage and tolvaptan is now available also in 7.5 mg tablets, since this dosage is becoming more frequently employed (38). The most common side effects of tolvaptan are dry mouth, thirst, increased urinary frequency, dizziness, nausea, and orthostatic hypotension (10). Another issue is the risk of sodium levels overcorrection. This event may be associated with osmotic demyelination syndrome (ODS). First described in 1959 by Adams and Victor (39), ODS embraces central pontine myelinolysis and extrapontine myelinosis, such as in the basal ganglia, cerebellar white matter, thalamus, or hippocampus (40). At histopathological evaluation, there is evidence of noninflammatory demyelination with simultaneous preservation of neurons and associated axons (41). To date, no published reports of osmotic demyelination syndrome occurring after a rapid increase during treatment with a vasopressin receptor antagonist are reported. Moreover, FDA issued a caution about hepatic injury that was noted in three patients who received tolvaptan in clinical trials examining its effect on autosomal dominant polycystic kidney disease (ADPKD) (42, 43).

Tolvaptan is effective in raising sodium levels in patients with euvolemic or hypervolemic hyponatremia as shown in the SALT-1 and SALT-2 studies (10), especially in case of hyponatremia secondary to SIAD (44). The American guidelines consider tolvaptan as a second-line treatment in case of fluid restriction failure (28). In US, the use of vaptans in SIAD could be considered in the treatment of mild-to-moderate hyponatremia and asymptomatic severe hyponatremia, whereas data on their use in symptomatic patients with profound hyponatremia are few. By contrast, the European guidelines generally recommend against the use of tolvaptan due to its potential risk of serum sodium levels overcorrection; however this phenomenon was seen more frequently in patients with sodium levels <125 mmol/L, as reported in the SALT studies (10) as well as in real-life practice (37).

Another promising therapeutic approach is represented by sodium-glucose co-transporter 2 (SGLT2)-inhibitors, such as empagliflozin, which was demonstrated to be effective in correcting hyponatremia in two recent randomized controlled trials (NCT02874807, NCT03202667) (45, 46).

In our patient, as mentioned above, we decided against water restriction, and it was not possible to administer either urea or demeclocycline. Although tolvaptan administration is not recommended by European guidelines, we decided to administer tolvaptan to our patient, which has been well tolerated until the last control. However, it was challenging to obtain a straight and long-term correction of serum sodium levels. Indeed, we administered tolvaptan in a once-daily dose initially at the lowest dosage (15 mg once a day), with the persistence of reduced serum sodium levels in repeated checks. Subsequently, the dosage was first doubled (30 mg once a day) and then tripled (45 mg once a day) with an initial improvement in serum sodium levels, but this was not maintained over time. We therefore tried dividing the 45 mg once-a-day tolvaptan dose into two daily doses of 30 mg at 8 am in the morning and 15 mg at 8 pm in the evening respectively, achieving serum sodium levels persistently in the low-normal range. These values were also confirmed at subsequent outpatient visits at three, six and nine months.

Tolvaptan half-life ranges between 6 to 8 hours in healthy subjects (47), and usually a once-daily administration is sufficient in correcting hyponatremia, as free water clearance values and consequently the rise of serum sodium levels remain stable after 12 and also 24-h tolvaptan administration (48). Only one study in the literature (26) performed a direct comparison between two different tolvaptan dosing regimens (30 mg once-daily and 15 mg twice-daily respectively, administered for 7 days) in patients with congestive heart failure. The rationale for this study was that, as expected, the drug effect tightly resembles its plasma concentrations, and with a once-daily administration, the majority of the effect on urine excretion rate (about 80%) is observed within the first 12 hours from tolvaptan administration. Therefore, the authors speculated that a twice-daily dosing regimen could produce a more balanced effect. However, the observed pharmacokinetic profile was similar, except for the Tmax (reached after 2 hours with the 30 mg once-daily regimen, and after ∼ 10 hours, just following the second dose, for the 15 mg twice-daily regimen) and the mean 24-hour AUC value (slightly higher with the 30 mg once-daily regimen). No clinically significant differences in any of the pharmacodynamic variables were detected. According to Hauptman et al., the lack of differences between the dosing regimens in the pharmacodynamic variables is due to the close similarity of the pharmacokinetic profiles (26). No studies have compared the pharmacokinetic and pharmacodynamic characteristics of tolvaptan in patients with SIAD so far.

As previously mentioned, CNS disorders can be a common cause of SIAD. CCA can be asymptomatic or present with cognitive deficits, muscle tone impairments and neuropsychological disorders (17). It can cause both hypernatremia secondary to central diabetes insipidus (18) or hyponatremia due to SIAD. To our knowledge, only two examples of CCA are currently reported in the literature as a cause of SIAD (22, 23), while seven cases of teratoma have been associated with SIAD (most of them improved after surgery) (49). Although we are aware that we cannot fully exclude the teratoma as a cause of anti-diuretic hormone hypersecretion (surgical removal was not performed, and a pathology report is not available), we believe that it can be hardly considered as the cause of SIAD in our patient, since only cases of immature ovarian teratoma associated with SIAD are reported in the literature. Furthermore, in our specific case a direct or indirect mechanical damage to the hypothalamus is unlikely because of its frontal localization. On the other hand, Meena et al. (22) described an 8-year-old girl with seizure disorder, severe hyponatremia (116 mmol/L) and concomitant CCA, while Silveira et al. (23) described a 41-year-old woman who presented with paresthesia in the right hand with low serum sodium levels (117 mmol/L) and a previous diagnosis of CCA. Our report is a rare case of chronic hyponatremia due to the SIAD associated with CCA. Probably, hyponatremia has not been previously diagnosed due to the overlapping symptoms determined by SIAD and CCA.

## 4 Conclusion

SIAD is a frequent cause of hyponatremia in hospitalized patients. The most frequent causes include cancers (such as small cell lung carcinoma), and diseases of the lung (such as pneumonia) or the central nervous system (such as subarachnoid hemorrhage). SIAD associated with CCA is a rare condition since only few cases are reported in the literature. Fluid restriction represents the first therapeutic line in SIAD, but different pharmacological agents are available, such as urea, demeclocycline or vasopressin receptor antagonists. Tolvaptan provides an effective therapeutic option for hyponatremia secondary to SIAD, especially when other strategies are not effective or feasible. Based on drug information leaflet, it is administered in a once-daily dose, but in case of hyponatremia with erratic serum sodium levels we showed that a twice-daily dosing can be more effective. Further studies on the pharmacokinetics and pharmacodynamics of vasopressin receptor antagonists in SIAD are therefore needed.

## Data availability statement

The original contributions presented in the study are included in the article/supplementary material. Further inquiries can be directed to the corresponding authors.

## Ethics statement

Written informed consent was obtained from the individual(s) for the publication of any potentially identifiable images or data included in this article.

## Author contributions

AA: Conceptualization, Visualization, Writing – original draft, Writing – review & editing. DD: Conceptualization, Writing – original draft. LD: Conceptualization, Writing – original draft. NM: Writing – review & editing. SG: Writing – review & editing. MB: Supervision, Writing – review & editing. DF: Supervision, Writing – review & editing. FG: Conceptualization, Supervision, Visualization, Writing – review & editing.
